# Beyond the brain-Peripheral kisspeptin signaling is essential for promoting endometrial gland development and function

**DOI:** 10.1038/srep29073

**Published:** 2016-07-01

**Authors:** Silvia León, Daniela Fernadois, Alexandra Sull, Judith Sull, Michele Calder, Kanako Hayashi, Moshmi Bhattacharya, Stephen Power, George A. Vilos, Angelos G. Vilos, Manuel Tena-Sempere, Andy V. Babwah

**Affiliations:** 1 Department of Cell Biology, Physiology & Immunology, Faculty of Medicine and Instituto Maimonides de Investigacion Biomedica de Córdoba (IMIBIC)/Hospital Reina Sofia, University of Córdoba, Avda. Menéndez Pidal s/n, Spain; 2 The Children’s Health Research Institute, London, Ontario, Canada; 3 Lawson Health Research Institute, London, Ontario, Canada; 4 Department of Obstetrics and Gynaecology, Division of Reproductive Endocrinology and Infertility, London, Ontario, N6C 2V5, Canada; 5 Department of Physiology, Southern Illinois University School of Medicine, Carbondale, IL 62901, USA; 6 Department of Physiology and Pharmacology, London, Ontario, N6C 2V5, Canada; 7 Department of Oncology, London, Ontario University of Western Ontario, London, Ontario, N6C 2V5, Canada; 8 CIBEROBN, Instituto de Salud Carlos III, 14004 Córdoba, Spain; 9 FiDiPro Program, Department of Physiology, University of Turku, 20520 Turku, Finland

## Abstract

Uterine growth and endometrial gland formation (adenogenesis) and function, are essential for fertility and are controlled by estrogens and other regulators, whose nature and physiological relevance are yet to be elucidated. Kisspeptin, which signals via Kiss1r, is essential for fertility, primarily through its central control of the hypothalamic-pituitary-ovarian axis, but also likely through peripheral actions. Using genetically modified mice, we addressed the contributions of central and peripheral kisspeptin signaling in regulating uterine growth and adenogenesis. Global ablation of *Kiss1* or *Kiss1r* dramatically suppressed uterine growth and almost fully prevented adenogenesis. However, while uterine growth was fully rescued by E2 treatment of *Kiss1*^−/−^ mice and by genetic restoration of kisspeptin signaling in GnRH neurons in *Kiss1r*^−/−^ mice, functional adenogenesis was only marginally restored. Thus, while uterine growth is largely dependent on ovarian E2-output via central kisspeptin signaling, peripheral kisspeptin signaling is indispensable for endometrial adenogenesis and function, essential aspects of reproductive competence.

Kisspeptins (KPs) are a group of peptides derived from KISS1, the primary product of the *KISS1* gene^1^,^2^,^3^,^4^,^5^. KPs signal via Gα_q/11_/β-arrestin-coupled KISS1 receptor (KISS1R)^2^,^6^,^7^,^8^ and the central KP/KISS1R signaling system is a potent trigger of hypothalamic gonadotropin-releasing hormone (GnRH) secretion and thereby a major positive regulator of the hypothalamic-pituitary-gonadal axis^9^,^10^. In addition, based on the expression of this signaling system at peripheral sites in healthy cells and tissues, kisspeptin signaling has also been proposed as a direct regulator of ovarian and testicular function, placentation, insulin secretion and kidney development^1^,^11^,^12^,^13^,^14^,^15^,^16^,^17^,^18^,^19^. Studies from the Babwah laboratory have also demonstrated that a functional kisspeptin signaling system is expressed in the mouse uterus on the luminal and glandular epithelia on the day of embryo implantation^20^,^21^ and provided compelling evidence through the use of the *Kiss1*^−/−^ and *Kiss1r*^−/−^ mice that extra-hypothalamic kisspeptin signaling potentiates embryo implantation^20^. Similarly, a recent study from the Tena-Sempere laboratory confirmed that while the re-expression of *Kiss1r* in GnRH neurons of *Kiss1r*^−/−^ mice is sufficient to reactivate the neuroendocrine axis and trigger full fertility, some gonadal functions were not completely restored in this rescued model^22^ suggesting the absence of peripheral kisspeptin signaling intrinsically perturbs gonadal physiology.

Mice with congenital ablation of the genes encoding kisspeptins (*Kiss1*^−/−^) or their receptor (*Kiss1r*^−/−^) exhibit hypogonadotropic hypogonadism and female mice display follicular development which stalls at the pre-antral and early antral stage; this results in infertility^10^,^23^,^24^. The observation that follicular development can advance to the antral stage likely reflects that FSH secretion is not completely abolished in these knockout (KO) mice^23^,^24^. While the follicles from these null mice maintain the capacity to produce and secrete 17β-estradiol (E2), *Kiss1*^−/−^ and *Kiss1r*^−/−^ mice do not exhibit the pre-ovulatory E2 surge^10^,^24^, and consequently follicles do not undergo final maturation and ovulation. In addition, despite near normal circulating levels of E2 in adult KO mice^10^,^24^, the KO uterus is significantly smaller than the WT uterus^10^,^20^,^24^,^25^,^26^, suggesting that other factors which stimulate uterine growth must be absent in mice congenitally devoid of kisspeptin signaling and/or that the uterus lacks ESR1 (estrogen receptor 1) expression in this model and hence is unresponsive to E2. However, an analysis of the pregnant uterus of *Kiss1r*^−/−^ mice indicated there was normal *Esr1* and ESR1 expression^20^. During the course of our investigations of the *Kiss1*^−/−^ and *Kiss1r*^−/−^ mice, we also noted that the KO uterus is almost completely devoid of endometrial glands^20^; an observation that is reminiscent of findings from a previous report by d’Anglemont de Tassigny *et al*.^24^, using an independently generated *Kiss1*^−/−^ mouse line (*Kiss1*^*tm1Coll*^).

Endometrial glands are found in all mammalian uteri where they produce and transport substances, such as leukemia inhibitory factor, that are required for the establishment of uterine receptivity and embryo implantation and survival^27^,^28^,^29^. In support of this, genetic inactivation of genes, such as *Foxa2, Wnt4* and *Wnt7a* that positively regulate endometrial gland formation (adenogenesis) result in subfertility and infertility^30^,^31^,^32^,^33^. In humans, endometrial adenogenesis begins in the fetus, continues postnatally, and is completed during puberty. In contrast, in sheep, pigs and rodents, adenogenesis typically begins during the early postnatal period and involves differentiation and budding of glandular epithelia from the luminal epithelium^29^,^31^. In mice, this begins around postnatal day (PND) 5–6 and is followed by extensive cell proliferation within the nascent glands (evident by PND7) leading to their elongation and invasion of the surrounding stroma^29^. The adult uterine histoarchitecture is established between PND14–21 with adenogenesis persisting over the lifespan^34^.

Initial adenogenesis and uterine growth in the neonate occur independently of ovarian and adrenal hormones in many species, including rodents^35^,^36^ and livestock^37^,^38^,^39^ as well as independently of ESR1 in pre-weaning mice^40^. On the other hand, elevation of ovarian hormones at the onset of puberty alters the mechanisms regulating adenogenesis and uterine growth, shifting them into an ovarian- and ESR1-dependent phase that begins around PND29 and lasts throughout adult life^34^,^40^.

In this scenario, it is intriguing that despite the fact that circulating E2 levels are reported to be near WT levels in the adult *Kiss1*^−/−^ and *Kiss1r*^−/−^ mice, these KO models exhibit markedly reduced uterine growth and adenogenesis. These observations would suggest that, in addition to E2, other peripheral factors (ovarian-derived or otherwise) driven by central and/or peripheral kisspeptin signaling and which are missing in these above kisspeptin null models, would physiologically contribute to uterine growth and endometrial adenogenesis. In this work, we aimed at elucidating this phenomenon using suitable genetically modified mouse models.

## Results

### Ablation of *Kiss1* or *Kiss1r* results in loss of adenogenesis and reduction in uterine growth in the adult female mouse

**Figure 1:**
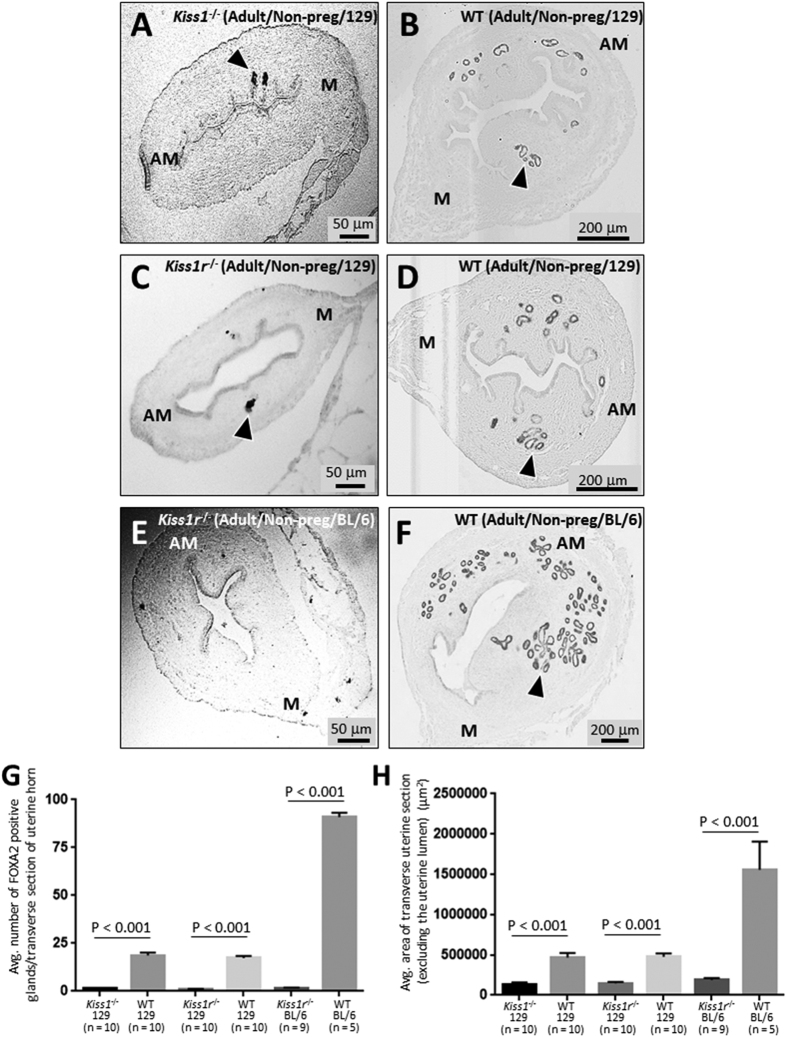
Ablation of *Kiss1* or *Kiss1r* results in loss of adenogenesis and reduction in uterine growth in the adult female mouse. Transverse uterine sections from adult (8–12 weeks old) non-pregnant 129S1/SvImJ *Kiss1*^−/−^ and WT littermate (**A**,**B**); 129S1/SvImJ *Kiss1r*^−/−^ and WT littermate (**C**,**D**) and C57BL/6J *Kiss1r*^−/−^ and WT littermate (**E**,**F**) were analyzed for FOXA2-positive endometrial glands and uterine size. Examples of FOXA2-positive glands are shown with arrowheads. Glands were quantified and uterine growth was determined by measuring the average area (μ^2^) of a transverse uterine section (excluding the uterine lumen) per uterine horn; data are displayed graphically (**G**,**H**). M: mesometrial; AM: anti-mesometrial. The number of independent investigations is reported in the figure and the data are shown as mean ± SEM.

The analysis of FOXA2 expression, a marker of endometrial glands, revealed that in transverse uterine sections from the *Kiss1*^−/−^ and *Kiss1r*^−/−^ (global KO) adult mice (8–12 weeks old, non-pregnant, 129S1/SvImJ genetic background), gland formation was reduced by about 93% vs. WT littermate controls (Fig. 1A–D,G). As an indication of uterine growth, the average area of transverse uterine sections was determined. In the *Kiss1*^−/−^ and *Kiss1r*^−/−^ mice, this index was found to be reduced by about 70% relative to that of respective WT littermates (Fig. 1A–D,H). The uterine phenotypes observed in *Kiss1*^−/−^ and *Kiss1r*^−/−^ 129S1/SvImJ mice were fully recapitulated in age-matched *Kiss1r*^−/−^ mice and WT littermates of the C57BL/6J genetic background (Fig. 1E–H), where gland formation in the *Kiss1r*^−/−^ mice was reduced by about 98% and the uterine area by about 78% compared to WT littermates (Fig. 1E,F,H). Our analyses also revealed that the endometrium of the C57BL/6J *Kiss1r* WT mouse contained about 5-fold more glands and the uterine area was about 3.3 times larger than age-matched 129S1/SvImJ WT *Kiss1* and *Kiss1r* mice (Fig. 1). Despite these strain differences, our convergent findings of the impact of lack of kisspeptin signaling on adenogenesis in these two mouse strains unambiguously demonstrate that kisspeptin signaling regulates uterine growth and development.

### E2 therapy partially rescues adenogenesis but fully rescues uterine growth in the pregnant adult *Kiss1* ^−/−^ mouse

Next, we sought to determine whether these striking uterine phenotypes in the *Kiss1*^−/−^ and *Kiss1r*^−/−^ mice might be due solely to insufficient E2 drive (the end-point of the central failure of the hypothalamic- pituitary-ovarian axis). Of note, although circulating E2 concentrations have been reported to be nearly similar between adult *Kiss1*^−/−^ and *Kiss1r*^−/−^ mice and their WT littermates^10^,^24^, the adult KO phenotypes are consistent with diminished ovarian function and E2 levels^34^. Furthermore, our recent results showed that chronic E2 therapy post-weaning coupled to gonadotropin treatment, rescued follicular development and triggered ovulation of fertilization-competent oocytes^20^. We therefore determined what effect E2 supplementation would have on the growth and development of the KO uterus. Since we were also interested in determining the impact of such treatment on early pregnancy, we examined uteri from E2-treated mice on D4 of pregnancy, the day on which embryo implantation occurs; a process dependent on glandular secretions^11^,^27^,^29^,^41^. Considering the commonalities in the phenotypes of the *Kiss1*^−/−^ and *Kiss1r*^−/−^ mice, these studies were conducted only in the *Kiss1*^−/−^ mouse.

**Figure 2:**
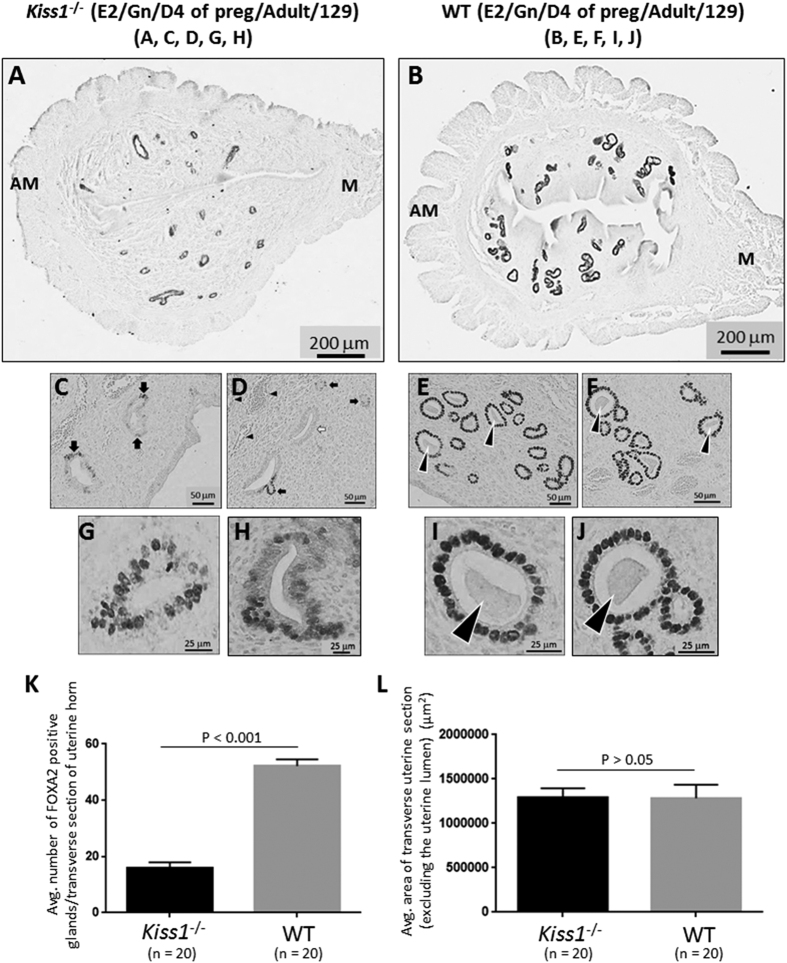
E2 therapy partially rescues adenogenesis but fully rescues uterine growth in the pregnant adult *Kiss1*^−/−^ mouse. Transverse uterine sections from E2- and gonadotropin-treated, adult (9–10 weeks old) pregnant 129S1/SvImJ *Kiss1*^−/−^ (**A,C,D,G,H**) and WT littermates (**B,E,F,I,J**) were analyzed for FOXA2-positive endometrial glands and uterine size. Glands were quantified and uterine growth was determined by measuring the average area (μ^2^) of a transverse uterine section (excluding the uterine lumen) per uterine horn; data are displayed graphically (**K,L**). M: mesometrial; AM: anti-mesometrial. Arrowheads in (**E**,**F**,**I**,**J**) show examples of glands with luminal secretions. The number of independent investigations is reported in the figure and the data are shown as mean ± SEM.

E2 administration for 5 weeks starting before puberty induced a significant increase in adenogenesis and uterine growth in both the adult KO and WT uterus (9–10 weeks old), as compared to closely age-matched (8–12 weeks old) adult untreated and non-pregnant KO and WT mice (Fig. 2A,B,K,L vs. Fig. 1A,B,G,H). Admittedly, part of this response would reflect the pregnant state that the mice were in and the other part the E2 treatment. However, despite 5 weeks of E2 administration and 4 days of pregnancy, the KO uterus still exhibited significantly diminished adenogenesis (Fig. 2A,B,K). Interestingly, however, while adenogenesis was only rescued by about 24%, uterine growth was fully restored compared to WT littermates (Fig. 2A,B,L). These results suggest that while central failure of the hypothalamic-pituitary-ovarian axis could be responsible for diminished uterine growth, it cannot solely account for severely reduced gland development, thereby implicating a role for peripheral kisspeptin signaling.

### E2-rescued glands in the *Kiss1* ^−/−^ endometrium exhibit diminished FOXA2 expression, hyperplasia of the glandular epithelium (GE) and a lack of secretion

Initial analyses of the *Kiss1*^−/−^ endometrium revealed that the majority of glands displayed a consistent decrease in FOXA2 expression (Fig. 2A,B). Additionally, in a smaller number of glands (about 20%), FOXA2 was detected only in a subset of cells comprising the GE (Fig. 2C–F) or not detected at all (Fig. 2C–F). In about 10% of the E2-rescued glands, the GE was comprised of a highly disorganized cellular layer that had undergone hyperplasia at one or more points (Fig. 2G,H); this was in striking contrast to the WT GE which was always comprised of a well-organized single layer of cells (Fig. 2I,J). While Stewart *et al*.^34^ reported that E2 administration to the neonate triggered a hyperplastic glandular phenotype in adult mice, it does not appear that the post-weaning-administration of E2 is the underlying cause of this hyperplasia since WT littermates were also E2-treated but did not display this phenotype (Fig. 2I,J). We also determined that *Ctnnb1* (β-catenin) expression was similar between the KO and WT littermates (*data not shown*) ruling out a deregulation of β-catenin in this phenotype^42^. Finally, it was observed that in all E2-rescued glands, the glandular lumen was completely devoid of any glandular secretions, while within a large number of WT glands secretions could be readily detected within the lumen (Fig. 2C–J; see arrowheads).

### E2-rescued glands in the *Kiss1* ^−/−^ endometrium appear non-functional

Given the finding that E2-rescued glands in *Kiss1*^−/−^ mice lack luminal secretions, we hypothesized that kisspeptin signaling positively regulates gland function (that is, the expression, secretion and transport of important regulators of implantation and decidualization) and that E2-rescued glands would be deficient in factors that regulate gland function. To test this idea, we first examined the expression of SPP1 (secreted phosphoprotein 1 also known as osteopontin). SPP1 is expressed in both the endometrial stroma and glands on D4 of pregnancy in the mouse and is suggested to positively regulate implantation^43^,^44^,^45^.

**Figure 3:**
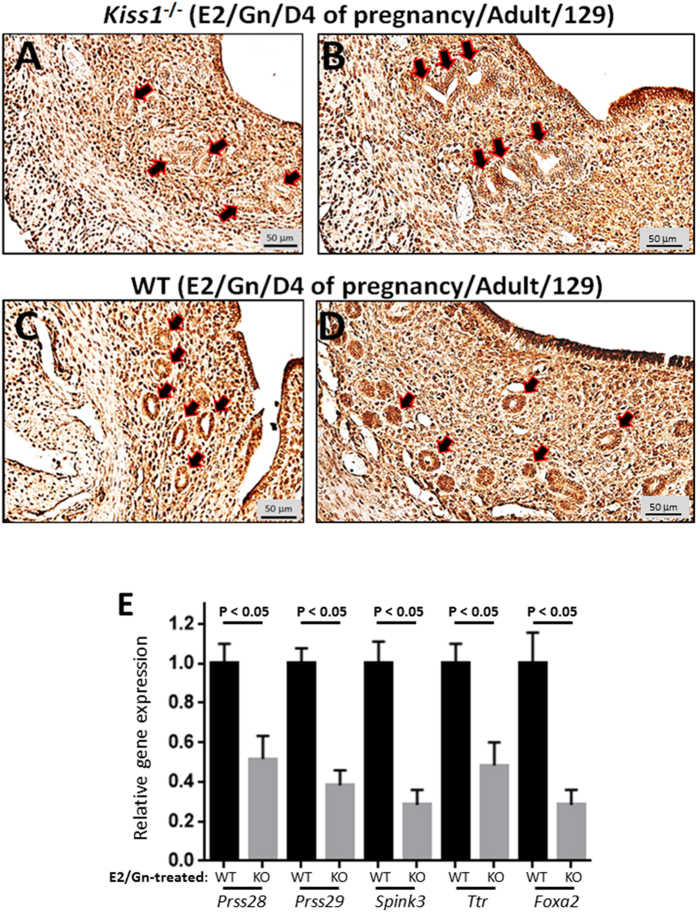
E2-rescued glands in the *Kiss1*^−/−^ endometrium are deficient in the expression of molecules that may play important roles in gland function. Transverse uterine sections from E2- and gonadotropin-treated, adult (9–10 weeks old) pregnant 129S1/SvImJ *Kiss1*^−/−^ (**A,B**) and WT littermates (**C,D**) were analyzed for SPP1 expression by immunohistochemistry. Experiment was conducted 3 independent times on uteri collected from mice of each genotype; representative sections from two mice of each genotype are shown. Whole uteri from E2- and gonadotropin-treated, adult (9–10 weeks old) pregnant 129S1/SvImJ *Kiss1*^−/−^ and closely age-matched WT littermate were analyzed for *Prss28, Prss29, Spink3, Ttr* and *Foxa2* expression by quantitative real-time RT-PCR (**E**). Quantitative RT-PCR was conducted 3 independent times on uteri collected from mice of each genotype and the data are shown as mean ± SEM.

Immunohistochemical analysis of the uteri from E2-treated *Kiss1*^−/−^ and WT littermates on D4 of pregnancy revealed that while SPP1 was expressed throughout the uterus in both KO and WT uteri, it was almost completely absent in the GE of all glands in the *Kiss1*^−/−^ endometrium (Fig. 3A–D). While SPP1 is expressed throughout the uterus, spatial examination allowed us to confirm that SPP1 expression was diminished in the GE. To continue testing our hypothesis, in the absence of well-validated antibodies, we quantified the expression of genes reported to be highly expressed (relative to other uterine cells) in the GE^46^; these were *Prss28* (protease, serine, 28)^47^, *Prss29* (protease, serine, 29)^47^, *Spink3* (serine peptidase inhibitor, Kazal type 3)^48^ and *Ttr* (transthyretin)^46^. As a positive control, *Foxa2* was also included in this analysis. Our results show that all genes including *Foxa2* exhibited a significant reduction in expression in E2-treated *Kiss1*^−/−^ mice (Fig. 3E) leading us to conclude that kisspeptin signaling directly regulates gland function.

### Re-expression of *Kiss1r* in the hypothalamus of adult *Kiss1r* ^−/−^ mice restores uterine growth and gland function, however, adenogenesis and FOXA2 expression are only partially rescued

To further explore the findings that central and peripheral kisspeptin signaling regulate gland development and function (Fig. 2), we conducted a complementary study where we compared gland number and uterine growth between adult *Kiss1r*^−/−^*Tg* mice and *Kiss1r*^−/−^ and WT littermates (C57BL/6J genetic background). The *Kiss1r*^−/−^*Tg* mice are *Kiss1r*^−/−^ mice in which *Kiss1r* is specifically re-expressed in GnRH neurons leading to a full reactivation of the neuroendocrine axis resulting in complete follicular maturation and ovulation and fertility equivalent to that of WT mice^22^,^26^.

**Figure 4:**
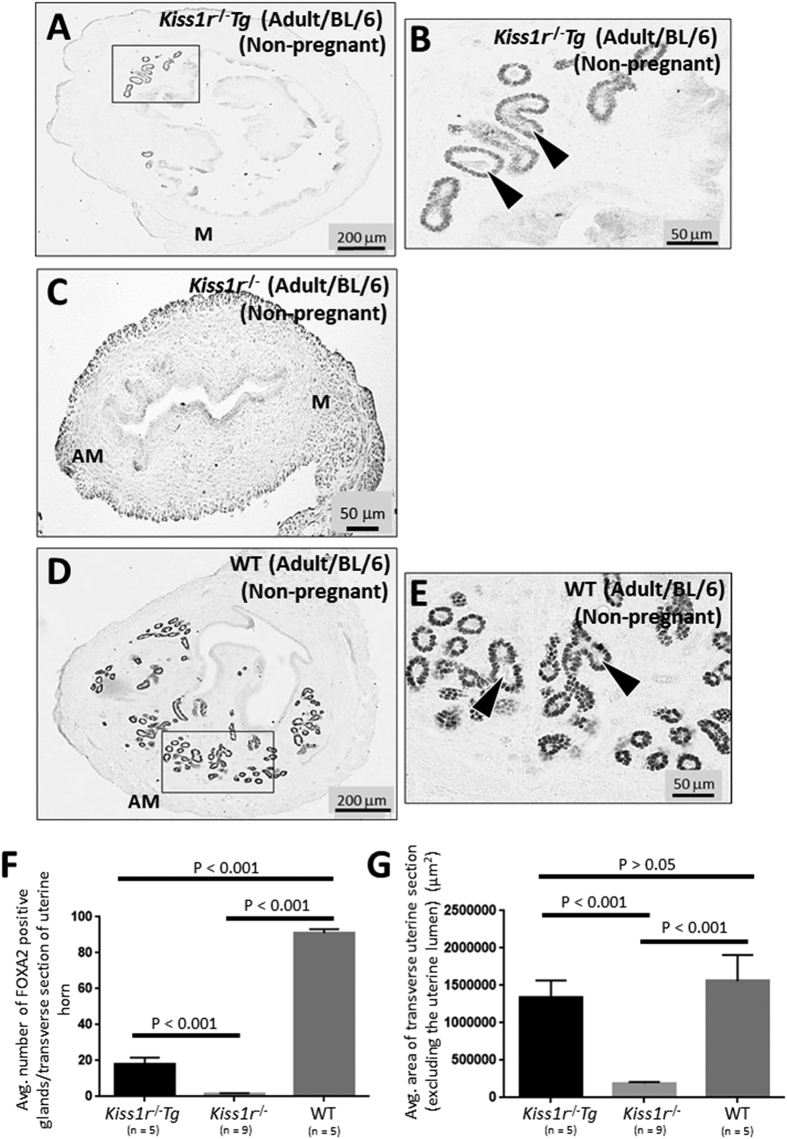
Re-expression of *Kiss1r* in the hypothalamus of adult *Kiss1r*^−/−^ mice restores uterine growth and gland function, however, adenogenesis and FOXA2 expression are only partially rescued. Transverse uterine sections from adult non-pregnant and aged-matched (8–12 weeks old) C57BL/6J *Kiss1r*^−/−^*Tg* mice (**A**,**B**) and *Kiss1r*^−/−^ (**C**) and WT littermates (**D**,**E**) were analyzed for FOXA2-positive endometrial glands and uterine size. Boxes in (**A,D**) are shown at higher magnification in (**B**,**E**). Glands were quantified and uterine growth was determined by measuring the average area (μ^2^) of a transverse uterine section (excluding the uterine lumen) per uterine horn; data are displayed graphically (**F**,**G**). Arrowheads in (**B**,**E**) show examples of glands with luminal secretions. M: mesometrial; AM: anti-mesometrial. The number of independent investigations is reported in the figure and the data are shown as mean ± SEM.

Adult *Kiss1r*^−/−^*Tg* mice contained significantly more glands than *Kiss1r*^−/−^ mice (Fig. 4A–C and F). However, the number of glands was significantly less than that observed in WT mice (*Kiss1r*^−/−^ littermates) (Fig. 4A,B,D–F). While FOXA2 was observed on almost every cell of the GE, signal intensity was visibly weaker than that in WT mice (Fig. 4A,B,D,E). Despite reduced adenogenesis and FOXA2 expression, the GE in the endometrium of the *Kiss1r*^−/−^*Tg* mice was comprised of a well-organized single layer of cells that was identical to the GE of WT mice (Fig. 4B,E). Additionally, the glandular lumen in the endometrium of the *Kiss1r*^−/−^*Tg* mice clearly displayed glandular secretions (Fig. 4B; see arrowheads). Regarding uterine growth, uteri from adult *Kiss1r*^−/−^*Tg* mice were significantly larger than *Kiss1r*^−/−^ but not different from WT mice (Fig. 4A,C,D,F,G). These results further strengthen the idea that while uterine growth is largely under the control of central kisspeptin signaling, both central and peripheral kisspeptin signaling regulate adenogenesis.

### Ovarian activin A output might be reduced in the *Kiss1r* ^−/−^ *Tg* mouse

Collectively, the studies conducted on the *Kiss1*^−/−^ and *Kiss1r*^−/−^*Tg* mice clearly reveal that in addition to E2, other peripheral factors that regulate adenogenesis are missing in these mice. Studies from the Spencer laboratory^49^,^50^ provided correlative data pointing out that components of the ovarian activin-follistatin system might regulate neonatal ovine uterine size and adenogenesis. These authors have also indicated the possibility that inhibins might be important regulators of these processes^50^. Therefore, we quantified the mRNA expression of the genes encoding follistatin (*Fst*) and the α subunit of inhibin (*Inha*) in the ovaries of *Kiss1r*^−/−^*Tg, Kiss1r*^−/−^ (global KO) and WT mice. We also examined the genes encoding the other subunits of inhibin A and activin A (βA: *Inhba*) and inhibin B and activin B (βB: *Inhbb*); activin AB being comprised of βA and βB subunits.

**Figure 5:**
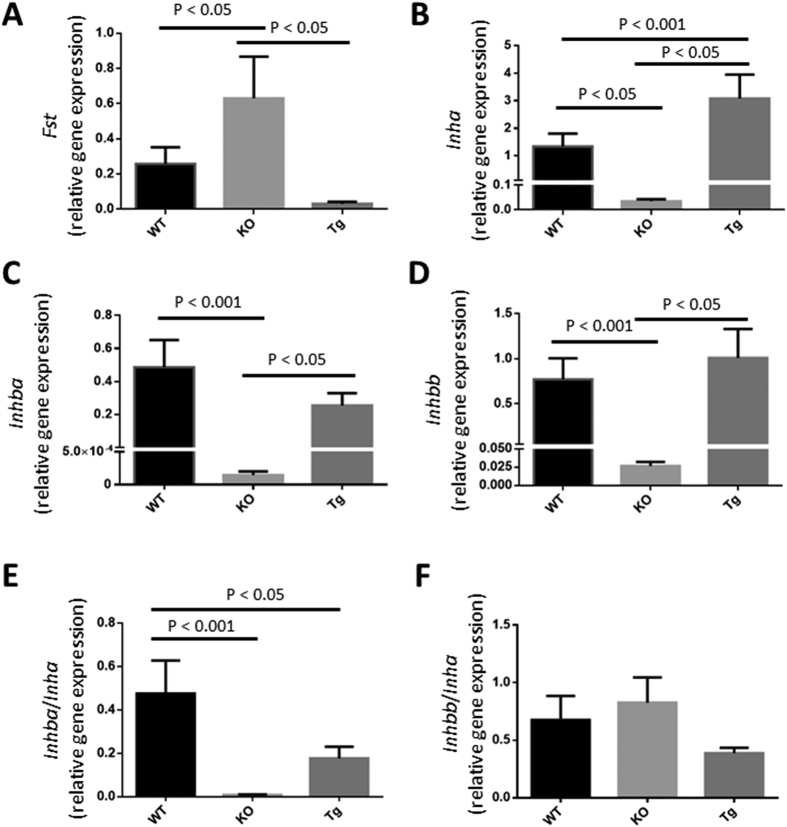
Ovarian activin A output might be reduced in the *Kiss1r*^−/−^*Tg* mouse. RNA isolated from the ovaries from adult non-pregnant and closely aged-matched (8–12 weeks old) C57BL/6J WT and *Kiss1r*^−/−^ (KO) littermates and *Kiss1r*^−/−^*Tg* (Tg) mice and were analyzed by quantitative RT-PCR for the expression of the genes encoding follistatin (*Fst*) (**A**) and the α subunit of inhibin (*Inha*) (**B**) and the other subunits of inhibin A and activin A (βA: *Inhba*) (**C**) and inhibin B and activin B (βB: *Inhbb*) (**D**). As a measure of activin (**A,B** and AB) output by the ovaries the *Inhba/Inha* (**E**) and *Inhbb/Inha* (**F**) ratios were calculated. Quantitative RT-PCR was conducted 3 independent times on ovaries (N = 4–6) collected from mice of each genotype.

Global loss of *Kiss1r* (KO) resulted in the significant up-regulation of ovarian *Fst* expression relative to WT littermates (Fig. 5A), while the reactivation of the neuroendocrine axis (*Kiss1r*^−/−^*Tg*) reduced expression to WT levels (Fig. 5A). As for *Inha*, there was a significant down-regulation in the KOs relative to WT littermates (Fig. 5B), while in *Kiss1r*^−/−^*Tg* mice expression was fully restored, reaching significantly greater levels than in the WT mice (Fig. 5B). The pattern of responses for *Inhba* and *Inhbb* were identical to each other (Fig. 5C,D) and similar to *Inha* (Fig. 5B), except that levels were not significantly different between WT and *Kiss1r*^−/−^*Tg* mice (Fig. 5C,D). Finally, we calculated the *Inhba/Inha* and *Inhbb/Inha* ratios as an indirect measure of activin (A, B and AB) output by the ovaries. The average *Inhba/Inha* ratio in the WT mouse was significantly greater than in the global KO, while *Kiss1r*^−/−^*Tg* mice displayed a partial rescue, although this parameter was markedly lower than in the WT and there was no significant difference vs. the ratio detected in the KO mouse (Fig. 5E). In contrast, *Inhbb/Inha* ratio was similar between the three genotypes (Fig. 5F). Therefore, the possibility exists that reduced levels of activin A might in part account for the reduced adenogenesis observed in the *Kiss1r*^−/−^*Tg* mouse (Fig. 4).

### Reduced hypothalamic GnRH secretion in the adult *Gnaq* ^*d/d*^ *;Gna11* ^−/−^ mouse reduces adenogenesis and uterine growth but triggers FOXA2 expression throughout the uterus

**Figure 6:**
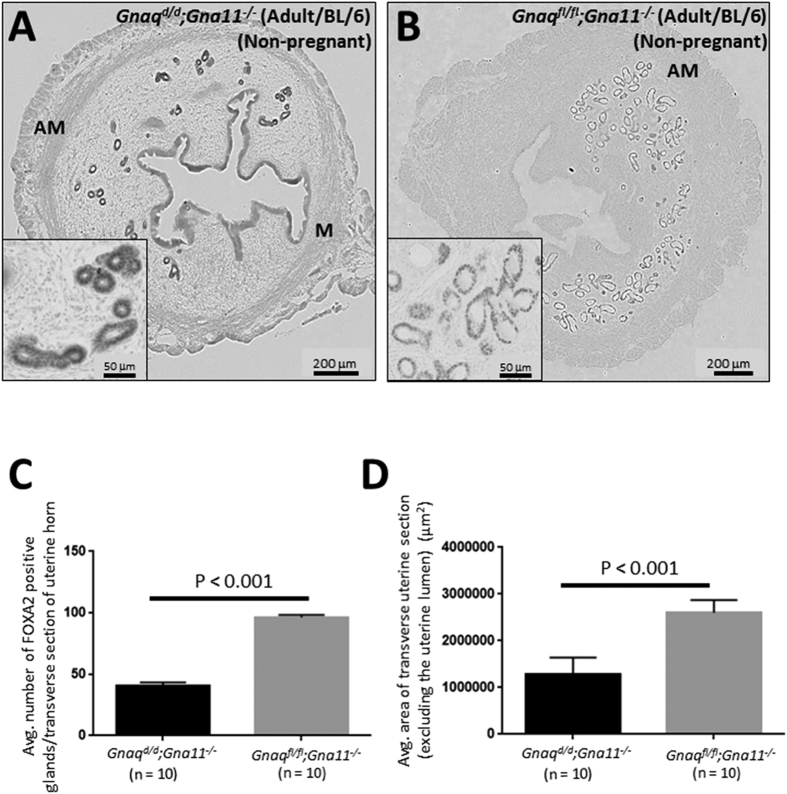
Reduced hypothalamic GnRH secretion in the adult *Gnaq*^*d/d*^*;Gna11*^−/−^ mouse reduces adenogenesis and uterine growth but triggers FOXA2 expression throughout the uterus. Transverse uterine sections from adult (8–12 weeks old) non-pregnant C57BL/6J *Gnaq*^*d/d*^*;Gna11*^−/−^ and *Gnaq*^*fl/fl*^*;Gna11*^−/−^ littermate controls were analyzed for FOXA2-positive endometrial glands and uterine size (**A**,**B**). Insets in (**A**,**B**) show parts of the endometrium at a higher magnification. Glands were quantified and uterine growth was determined by measuring the average area (μ^2^) of a transverse uterine section (excluding the uterine lumen) per uterine horn; data are displayed graphically (**C**,**D**). M: mesometrial; AM: anti-mesometrial. The number of independent investigations is reported in the figure and the data are shown as mean ± SEM.

The *Gnaq*^*d/d*^*;Gna11*^−/−^ mouse (C57BL/6J genetic background) conditionally lacks Gα_q/11_-signaling in its GnRH neurons and is significantly, though not completely, compromised in its ability to trigger kisspeptin-dependent GnRH secretion from the hypothalamus^7^. However, unlike the *Kiss1*^−/−^, *Kiss1r*^−/−^ and *Kiss1r*^−/−^*Tg* mouse, peripheral kisspeptin signaling is preserved. Analysis of the *Gnaq*^*d/d*^*;Gna11*^−/−^ uterus, relative to that of *Gnaq*^*fl/fl*^*;Gna11*^−/−^ littermate controls, revealed that gland number and uterine growth were significantly reduced; findings consistent with the idea that the central axis regulates adenogenesis and uterine growth (Fig. 6). However, although gland number and uterine growth were significantly reduced by about 58% and 51%, respectively, the *Gnaq*^*d/d*^*;Gna11*^−/−^ uterus still exhibited greater adenogenesis and uterine growth than the untreated *Kiss1*^−/−^ and *Kiss1r*^−/−^ mouse lines, where adenogenesis was almost completely ablated and uterine growth reduced by about 81% (Fig. 1A–H). Although this smaller reduction might be partially due to the fact that these mice still exhibit central kisspeptin signaling, though greatly diminished^7^, it is highly probable that this milder phenotype is also caused by the fact that, in contrast to *Kiss1* and *Kiss1r* null models, they retain peripheral kisspeptin signaling intact.

Finally, and unexpectedly, FOXA2 expression was visibly and consistently increased in the GE of all glands in the *Gnaq*^*d/d*^*;Gna11*^−/−^ uterus as well as throughout the rest of the uterus, in particular the luminal epithelium (Fig. 6A,B). Elevated glandular and ectopic FOXA2 expression was never observed in the *Kiss1*^−/−^, *Kiss1r*^−/−^ or *Kiss1r*^−/−^*Tg* mouse of similar age (Fig. 1). Since the major difference between the *Gnaq*^*d/d*^*;Gna11*^−/−^ mouse and the *Kiss1*^−/−^, *Kiss1r*^−/−^ and *Kiss1r*^−/−^*Tg* mouse is that *Kiss1* and *Kiss1r* continue to be expressed peripherally in the *Gnaq*^*d/d*^*;Gna11*^−/−^ mouse, the data would strongly implicate peripheral kisspeptin signaling as the driving force behind the increased glandular and ectopic FOXA2 expression in the uterus of the adult *Gnaq*^*d/d*^*;Gna11*^−/−^ mouse.

### Adenogenesis and uterine growth are disrupted in the juvenile *Kiss1* ^−/−^ mouse

**Figure 7:**
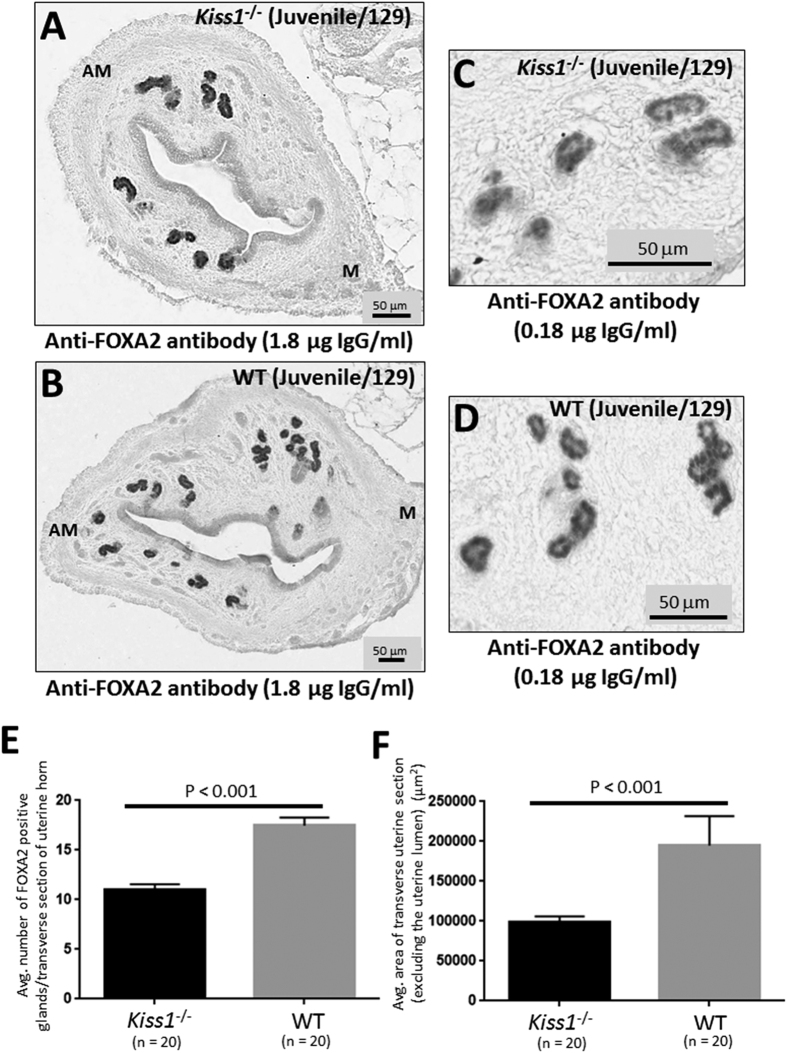
Adenogenesis and uterine growth are disrupted in the juvenile *Kiss1*^−/−^ mouse. Transverse uterine sections from juvenile (3 weeks old) 129S1/SvImJ *Kiss1*^−/−^ (**A,C**) and WT littermates (**B,D**) were analyzed for FOXA2-positive endometrial glands and uterine size. Sections shown in (**A**,**B**) were analyzed using the anti-FOXA2 antibody at a concentration of 1.8 μg IgG/ml. while those in (**C**,**D**) were analyzed using anti-FOXA2 antibody at a concentration of 0.18 μg IgG/ml. Glands were quantified and uterine growth was determined by measuring the average area (μ^2^) of a transverse uterine section (excluding the uterine lumen) per uterine horn; data are displayed graphically (**E,F**). M: mesometrial; AM: anti-mesometrial. The number of independent investigations is reported in the figure and the data are shown as mean ± SEM.

While our data revealed that kisspeptin signaling regulates adenogenesis and uterine growth, at both central and peripheral levels, the question that remained was whether kisspeptin signaling regulates adenogenesis and uterine growth in the juvenile mouse, a developmental period in which these processes are thought to occur in an adrenal- and ovarian-independent manner^34^,^36^,^40^. We therefore examined gland number and uterine growth in juvenile (PND21) *Kiss1*^−/−^ mice and their WT littermates. Results clearly showed that in juvenile *Kiss1*^−/−^ mice adenogenesis and uterine growth were significantly diminished (Fig. 7A,B,E,F), demonstrating that kisspeptin regulates these processes in the juvenile mouse; a phenomenon that, according to previous evidence, should occur independently of the ovaries. Of note, while loss of *Kiss1* significantly reduced gland formation and uterine growth in the juvenile mouse, relative to WT littermates, these parameters were only reduced by about 37 and 49%, respectively, therefore suggesting the roles for other signaling pathways in regulating these ovarian-independent processes in the juvenile mouse.

When FOXA2 expression was first inspected in the juvenile uteri from *Kiss1*^−/−^ and WT littermates, it was clear that FOXA2 expression was more intense on the glands in both the juvenile KO and WT uteri compared to that seen in the adult *Kiss1*^−/−^, *Kiss1r*^−/−^, *Kiss1r*^−/−^*Tg* and *Gnaq*^*d/d*^*;Gna11*^−/−^ mice and their respective WT controls (Fig. 7 vs. Figs 1,2,4 and 6). Importantly, in all studies FOXA2 was detected under identical conditions. Because the expression level was so intense in the juvenile period, we could not initially assess whether there was a difference in glandular expression between the KO and WT mice. The study was therefore repeated using the anti-FOXA2 antibody at a 10-fold dilution (0.18 μg/ml IgG). Results show that, under these conditions, glandular FOXA2 levels did not appear strikingly different between *Kiss1*^−/−^ and WT littermates (Fig. 7C,D).

## Discussion

**Figure 8:**
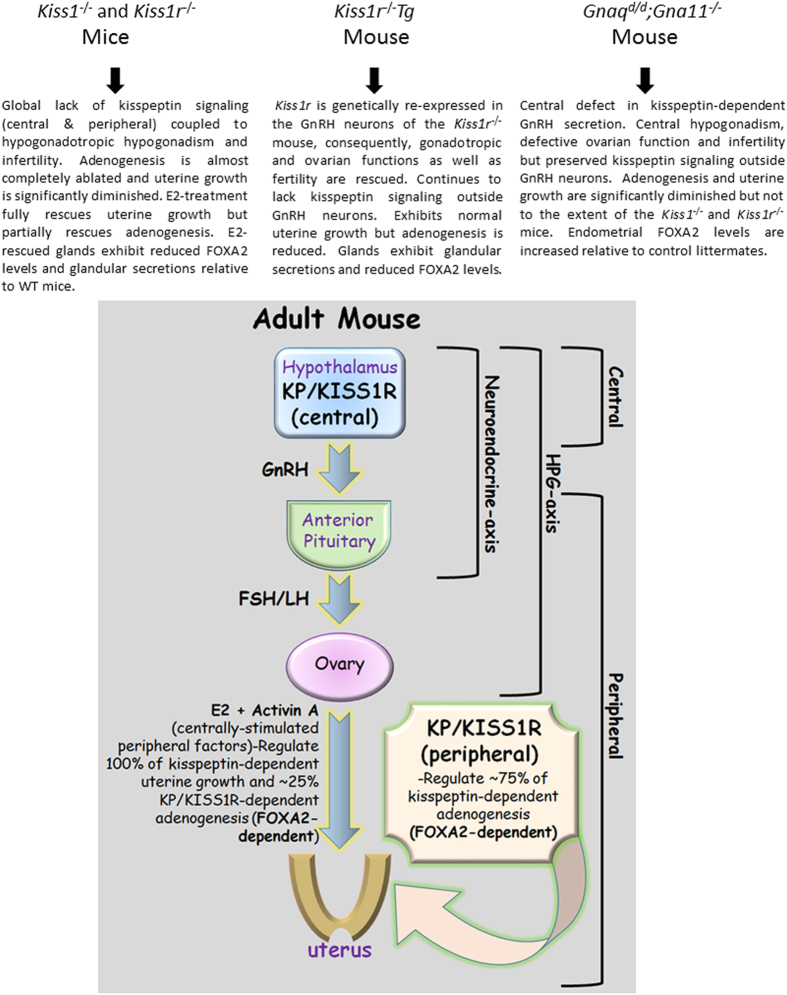
Phenotypic summary of the *Kiss1*^−/−^, *Kiss1r*^−/−^, *Kiss1r*^−/−^*Tg* mice *Gnaq*^*d/d*^*;Gna11*^−/−^ mice and cartoon illustrating the central and peripheral kisspeptin/KISS1R signaling pathways that potentiate endometrial gland development in the adult female mouse. It is important to note that while activin A might be one of the centrally-stimulated peripheral factors that induces kisspeptin-dependent adenogenesis, other factors might also exist. Additionally, although there is unambiguous evidence that kisspeptin-dependent adenogenesis is positively regulated by peripheral kisspeptin signaling, where this signaling is localized is currently unknown.

Our study reveals that global inactivation of *Kiss1* or *Kiss1r* results in an almost complete loss (about 97%) of total endometrial gland formation and a significant reduction (about 81%) in uterine growth in the adult mouse. Therefore, the kisspeptin signaling system must be a major regulator of adenogenesis and uterine growth in the adult mouse. Since E2 treatment of the *Kiss1*^−/−^ mouse and re-expression of *Kiss1r* in GnRH neurons in the *Kiss1r*^−/−^ mouse restored uterine growth fully but adenogenesis only by about an average of 25%, our results document a striking dissociation in the kisspeptin-dependent pathways controlling these two related, but clearly distinct phenomena. Thus, while uterine growth in the adult is largely dependent on central kisspeptin signaling, endometrial adenogenesis is predominantly regulated by peripheral kisspeptin signaling, which accounts for about an average of 75% of kisspeptin-dependent gland development (Fig. 8). These findings are further reinforced by the data from the *Gnaq*^*d/d*^*;Gna11*^−/−^ strain, a conditional KO that exhibits diminished central kisspeptin signaling but fully preserved peripheral kisspeptin actions, in which the reduction in adenogenesis and uterine growth was smaller than that seen in *Kiss1* and *Kiss1r* null mice (Fig. 8).

Taken together, the studies conducted on the E2-treated *Kiss1*^−/−^ and *Kiss1r*^−/−^*Tg* (rescued) mice revealed that in addition to E2, other centrally stimulated ovarian factors are needed for endometrial adenogenesis. These could include progesterone, follistatin, inhibins and activins. It is also possible that, at the dose set, the exogenously administered E2 was insufficient for triggering maximal adenogenesis. Of note, follistatin, activins, and inhibins regulate growth and differentiation of many branched epithelia-mesenchymal organs^51^,^52^, and have been suggested to play a role in uterine growth and adenogenesis in ovine neonates^49^,^50^. Our analyses in the global *Kiss1*^−/−^ and the *Kiss1r*^−/−^*Tg* mice strongly suggest that activin A, whose output, measured as *Inhbb/Inhba* ratio, is severely blunted in the global KO and significantly reduced in the rescued model, might co-operate with E2 in the control of kisspeptin-dependent uterine growth and development (Fig. 8). In addition, reduced activin B and inhibin, and/or increased follistatin production might contribute to the severe uterine phenotype of mice with global inactivation of kisspeptin signaling.

A number of genes are known to positively regulate adenogenesis in the mouse uterus. These include *Wnt4*^32^, *Wnt7a*^31^,^33^, *Wnt5a*^53^, *Ctnnb1* (β-catenin)^42^,^54^,^55^, *Foxa2*^30^, *Hoxa11*^56^, *Dicer1*^57^,^58^, *Lgr4*^59^, *Dlx5* and *Dlx6*^60^. In each case, conditional inactivation of these genes in the uterus resulted in an almost complete loss of gland formation. While the majority of these studies only examined adenogenesis in the adult mouse or shortly after weaning (PND21) and reported on a severe reduction in gland number compared to control mice, adenogenesis was also examined in the neonatal and juvenile periods in mice lacking *Dicer* in Müllerian duct mesenchyme-derived tissues of the reproductive tract and in the juvenile period in mice lacking *Lgr4* in epithelial cells^59^. In the case of the conditional *Dicer* KO, initial adenogenesis at PND4 and 8 was similar between the conditional KO and control mice but by PND14 and 21 glands were almost absent^57^,^58^. However, from about 5 weeks to 4 months of age the number increased gradually but at all times was consistently reduced compared to control mice. In the conditional *Lgr* KO, glands were also almost completely absent at PND21 and this remained unchanged at 9 weeks of age^59^. Essentially, the same finding was made with the *Kiss1* KO in the juvenile period (PND21), except that at PND21 a greater number of glands was detected in the *Kiss1*^−/−^ endometrium. Taken together, it appears that *Kiss1, Dicer* and *Lgr4* are important regulators of adenogenesis in the juvenile period, which is characterized as being ovarian- and ESR1-independent.

Our study revealed that in the adult E2-treated *Kiss1*^−/−^ mouse and the *Kiss1r*^−/−^*Tg* mouse not only was gland number reduced but so was FOXA2 levels on the GE. This striking phenotype led us to conclude that kisspeptin signaling regulates adenogenesis in a FOXA2-dependent manner. Based on this, it was predicted that FOXA2 expression would have also been reduced in the *Gnaq*^*d/d*^*;Gna11*^−/−^ mouse, but surprisingly the opposite was seen. Perhaps, this reflects a peripherally-stimulated compensatory response to diminished central signaling in this mouse. Why then was there not a similar central response to absent peripheral signaling in adult E2-treated *Kiss1*^−/−^ and *Kiss1r*^−/−^*Tg* mice? The answer to this interesting question is not known and might be linked to the observation that central signaling only accounts for about 25% of all adenogenesis while peripheral signaling accounts for the rest. More importantly, this observation further reinforces that mechanistically, central and peripheral kisspeptin pathways regulate adenogenesis differently.

This putative relationship between kisspeptin signaling and FOXA2 was only uncovered through our ability to rescue adenogenesis in suitable models, such as the E2-treated *Kiss1*^−/−^ and the *Kiss1r*^−/−^*Tg* mouse, before assessing FOXA2 expression. FOXA2 belongs to a family of three forkhead transcription factors encoded by different genes and is implicated in the development of organs such as the liver, pancreas, lung, prostate and uterus^61^,^62^,^63^,^64^. In the uterus, FOXA2 is uniquely localized to the GE in the WT endometrium and is essential for adenogenesis in the mouse^30^,^46^. Recently, Filant *et al*.^46^ undertook a genome-wide investigation of *in vivo* FOXA2 binding target regions in the neonatal and adult uterus and found that in the neonatal uterus, FOXA2-bound genes in the GE were enriched for developmentally related processes including cell cycle, cell junction and focal adhesion while in the adult uterus there was an enrichment for functional processes including metabolic pathways, focal adhesion and WNT signaling. These important results further define how FOXA2 regulates endometrial gland development and function.

In our initial characterization of the infertility observed in *Kiss1*^−/−^ and *Kiss1r*^−/−^ mice, we found that LIF was absent in all endometrial glands of E2-treated KO mice but if given exogenously could rescue the implantation defect^20^. This initially led us to conclude that LIF lies downstream of kisspeptin and that kisspeptin signaling is a positive regulator of glandular LIF expression and secretion. However, we now realize that diminished LIF expression is the indirect consequence of having non-functional glands and that it is less likely that kisspeptin signaling positively regulates its expression. This conclusion is based on the findings that (1) FOXA2 expression is diminished in glands from the *Kiss1r*^−/−^*Tg* mouse and sometimes even absent in the E2-rescued *Kiss1*^−/−^ mouse; (2) normal glandular morphology is disrupted in the E2-rescued *Kiss1*^−/−^ mouse; and (3) glands in the E2-rescued *Kiss1*^−/−^ mouse exhibit diminished expression of FOXA2^30^, LIF^27^, SPP1^43^,^44^,^45^, *Prss28*^47^, *Prss29*^47^, *Spink3*^48^ and *Ttr*^46^, molecules implicated in gland development and function^46^. We therefore suggest that kisspeptin signaling positively regulates both gland development and function and that in E2-treated *Kiss1*^−/−^ mice, while development is partially rescued, function is not. Interestingly, both development and function appeared to have been partially rescued in the *Kiss1r*^−/−^*Tg* mouse, again highlighting that other centrally-stimulated peripheral factors were missing in the E2-treated *Kiss1*^−/−^ mouse.

The data presented in this study reveal that kisspeptin-dependent adenogenesis is regulated by both central and peripheral pathways, but it is unknown whether both pathways contribute to the development of a single pool of glands or whether each contributes to a discrete pool. Additionally, while it is established that the central system resides in the hypothalamus, it remains unknown where the peripheral signaling system actually resides (Fig. 8). Based on a description of peripheral cells and tissues that express either kisspeptins and/or their receptors, possible sites are the ovary and uterus, although the contribution of other non-reproductive sites of action of kisspeptins, such as the liver and pancreas, cannot be excluded^1^,^13^,^16^,^18^,^20^. Although yet to be fully proven, we suggest that the uterus remains a strong candidate given our previously published data showing that on D4 of pregnancy, the uterus expresses a functional kisspeptin signaling system, on both the luminal and glandular epithelia^20^,^21^.

All mammalian uteri contain endometrial glands that secrete substances that positively regulate embryo implantation and subsequently support the survival and development of the conceptus (embryo and associated placental membranes) during pregnancy^28^,^29^. Human uterine secretions are enriched in cytokines, chemokines and growth factors and their levels appear to correlate positively with successful implantation and the establishment of a chemical pregnancy^65^,^66^,^67^. Despite these important findings, our understanding of gland function in human pregnancies lags behind our understanding in laboratory and domestic animals. Therefore, studies such as those described here with the *Kiss1*^−/−^, *Kiss1r*^−/−^, *Kiss1r*^−/−^*Tg* the *Gnaq*^*d/d*^*;Gna11*^−/−^ mouse will allow us to develop and test hypotheses designed to better understand gland function in human pregnancies. A better understanding could lead to higher implantation rates and successful pregnancy outcomes following assisted reproduction. Based on the current study, we conclude that while uterine growth in the adult is largely dependent on central kisspeptin signaling, endometrial adenogenesis is predominantly regulated by peripheral kisspeptin signaling, which accounts for about 75% of kisspeptin-dependent gland development (Fig. 8).

## Methods

### Mice

The *Kiss1*^*tm1Rla*^ (*Kiss1*^−/−^) and *Kiss1r*^*tm1Rla*^ (*Kiss1r*^−/−^) mice are global knockouts generated in the 129S1/SvImJ genetic background, and are generous gifts to Dr. A. V. Babwah from Dr. S.B. Seminara (Massachusetts General Hospital, Boston, Massachusetts, USA)^23^. Since the homozygous *Kiss1*^−/−^ and *Kiss1r*^−/−^ mice are infertile, each genotype was generated by mating heterozygous males to heterozygous females. These matings produced a segregating population of homozygous, heterozygous and WT littermates. Genotypes were identified as previously described^20^.

The *Kiss1r*^−/−^*Tg* (also referred to as *Gpr54*^−/−^*Tg*) mouse is a GnRH neuronal-specific *Kiss1r* expressing (rescued) mouse line generated in the C57BL/6 background using BAC transgenesis, and is a generous gift to Dr. M. Tena-Sempere from the groups of Drs. G. Schuzt and M. Kirilov (German Cancer Research Center, Heidelberg, Germany) and A.E. Herbison (Centre of Neuroendocrinology, University of Otago, NZ)^26^. *Kiss1r*^−/−^*Tg* mice are fertile, and the line was maintained by crossing *Kiss1r*^−/−^*Tg* males to females.

*Kiss1r*^−/−^ in the C57BL/6 background was also obtained from Drs. G. Schuzt, M. Kirilov and A. E. Herbison. The C57BL/6 *Kiss1r*^−/−^ mouse was generated independently from the 129S1/SvImJ *Kiss1r*^*tm1Rla*^ (*Kiss1r*^−/−^) mouse described above. Since homozygous C57BL/6 *Kiss1r*^−/−^ mice are also infertile, they were generated by mating heterozygous males to heterozygous females. These matings produced a segregating population of homozygous, heterozygous and WT littermates. Genotypes were identified as previously described^22^.

The *Gnaq*^*d/d*^*;Gna11*^−/−^ mouse, which was created in the Babwah laboratory^7^, is a global knockout for *Gna11* but conditionally lacks *Gnaq* in its GnRH neurons. Consequently, Kiss1r-coupled Gα_q/11_-signaling at the level of the GnRH neuron is abolished but Kiss1r continues to signal and mediate kisspeptin-dependent GnRH secretion, albeit weakly, via the β-arrestin-dependent pathway^7^. The mouse was generated in the C57BL/6J genetic background and is infertile. Therefore, the *Gnaq*^*d/d*^*;Gna11*^−/−^ mouse and *Gnaq*^*fl/fl*^*;Gna11*^−/−^ littermate controls were generated by crossing the *Gnaq*^*fl/fl*^*;Gna11*^−/−^ line to a line bearing the GnRH-Cre transgene and the segregating genotypes were identified as previously described^7^.

### Animal husbandry

Animal studies involving the *Kiss1*^−/−^ and *Kiss1r*^−/−^ mice and their WT littermates (129S1/SvImJ genetic background) and the *Gnaq*^*d/d*^*;Gna11*^−/−^ mouse and its littermate controls were approved by the University of Western Ontario Animal Care Committee according to guidelines established by the Canadian Council on Animal Care. Animal studies involving the *Kiss1r*^−/−^*Tg (Gpr54*^−/−^*Tg*) mouse and controls (*Kiss1r*^−/−^ and WT littermates on the C57BL/6 background) were approved by the Córdoba University Ethical Committee of animal experimentation and conducted in accordance with the European Union guidelines for use of experimental animals. In all cases, mice were maintained under a 12 h light/dark cycle and provided with standard rodent chow and water ad libitum.

### Hormonal treatments

Three to four week-old female mice (*Kiss1*^−/−^ and WT littermates) were administered E2 (100 μg/100 μl sesame oil) subcutaneously every 3–4 days over a 5-week period, then administered 7.5 IU pregnant mare serum gonadotropin (PMSG; Folligon; Intervet) intraperitoneally (i.p.) followed 48 hours later by 7.5 IU human chorionic gonadotropin (hCG; Chorulon; Intervet) i.p. Immediately after the hCG injection, mice were mated to WT males (D0 = day of mating)^20^. On D4 of pregnancy, uteri were collected and glands were characterized by analyzing FOXA2 and SPP1 immunoreactivity. The D4 uteri were used in quantifying the mRNA levels of *Prss28, Prss29, Spink3, Ttr* and *Foxa2*.

### Quantitative real-time RT-PCR studies

Gene expression studies were conducted independently in the Babwah and Tena-Sempere Laboratories. Protocols employed by each laboratory are described below.

#### Babwah Laboratory (for the analysis of uterine Foxa2, Prss28, Prss29, Spink3 and Ttr)

**Gene expression was determined on total RNA prepared from the entire uterine horns of experimental and control mice. Freshly harvested tissues were collected in RNAlater (Life Technologies Inc., Burlington, ON, Canada) and RNA was isolated using the Qiagen RNeasy mini kit according to manufacturer’s instructions (Qiagen, Missassauga, ON, Canada). One μg of total RNA was reverse-transcribed using SuperScript II (Invitrogen, Burlington, ON, Canada). Reactions were performed according to the manufacturer’s protocol using random hexamer primers (Amersham, Piscataway, NJ). Quantitative real-time PCR was performed in duplicate for each sample and done a total of three independent times using IQ SYBR Green Master Mix (Bio-Rad Laboratories, Mississauga, ON, Canada). To determine PCR efficiency, a 10-fold serial dilution of cDNA was performed as described previously^68^. Gene expression was normalized to *Actb* expression and presented as relative expression using the Pfaffl method^69^. Expression of the following genes was quantified using the following primers (presented 5′-3′). *Foxa2*-F: AGCAGAGCCCCAACAAGA and *Foxa2-*R: AGAGAGAGTGGCGGATGGAG (RefSeq ID: NM_010446.3); *Prss28*-F: CATCCGACGAGCACAAAG and *Prss28*-R: CCCAGAGTCACCAAAA CAG (RefSeq ID: NM_053259.2); *Prss29*-F: GTCAAGCTGCCCTCTGAGTC and *Prss29*-R: TGGTTG CCTGCACATAACAT (RefSeq ID: NM_053260.3); *Spink3*-F: AACGCATAGAGCCTGTCCT and *Spink3*-R: ACGAACCCACTTGCCAAA (RefSeq ID: NM_009258.5); *Ttr*-F: CAGAGTGGACCAACCG and *Ttr*-R: CCCAGGGCTTTTGAACATGC (RefSeq ID: NM_013697.5); *Actb*-F: TTCTACAATGAGCTGCGTGTG and *Actb*-R: GGGGTGTTGAAGGTCTCAAA (RefSeq ID: NM_007393.5).

#### Tena-Sempere Laboratory (for the analysis of ovarian Fst, Inha, Inhba and Inhbb)

Total RNA was extracted using TRIsure isolation reagent (Bioline Reagents Ltd., UK) and treated with DNase Q1 (Promega corporation, USA). One μg of total RNA was subjected to reverse transcription using IScript cDNA Synthesis kit (Bio-Rad Laboratories Inc., USA). For real-time PCR, we used Go Taq qPCR Master mix (Promega Corporation, USA) in a CFX96 Touch Real-Time PCR Detection System (Bio-Rad Laboratories Inc., USA). Primer-specific amplification and quantification cycles were run at 95 °C for 25 s, 62 °C for 25 s and 72 °C for 30 s and a final extension of 72 °C for 20 s. To normalize the quantification of inhibins subunits and follistatin mRNA level, we measured the amount of ribosomal 18S mRNA in each protocol. The corresponding standard curve for each gene was obtained by serial dilution of a reference ovarian cDNA sample. Expression of the following genes was quantified using the following primers (presented 5′-3′). *Inha*-F: CCTTTTGCTGTTGACCCTACG and *Inha-*R: AGGCATCTAGGAATAGAGCCTTC (RefSeq ID: NM_010564.4); *Inhba*-F: CTTCGTCTCTAATGAAGG CAACC and *Inhba*-R: CTCCACCACATTCCACCTGTC (RefSeq ID: NM_008381.3); *Inhbb*-F: GGA GAACGGGTATGTGGAGA and *Inhbb*-R: TGGTCCTGGTTCTGTTAGCC (RefSeq ID: NM_008380.1); *Follistatin*-F: AAAACCTACCGCAACGAATG and *Follistatin*-R: TTCAGAAGAGGA GGGCTCTG (RefSeq ID: NM_010565.3).

### Immunohistochemistry

Uteri were collected and processed for paraffin immunohistochemistry, as described previously^20^. Sections were then incubated in rabbit anti-FOXA2 IgG (1.8 μg/ml, catalogue # AB108422, ABCAM, Cambridge, MA, USA) or rabbit anti-SPP1 IgG (1:10,000 dilution, catalogue number AB10910, Millipore, Etobicoke, ON, Canada). In experiments represented by Fig. 7C,D, anti-FOXA2 IgG was used at a final concentration of 0.18 μg/ml. Antigen-bound primary antibodies were detected with the ImmunoCruz rabbit ABC Staining System (catalogue number sc-2018, Santa Cruz Biotechnology, Inc. Dallas, TX, USA). The secondary detection systems were used according to the manufacturers’ guidelines without any adaptations. Experimental and control samples were processed in parallel and treated with the 3,3′-diaminobenzidine substrate for an identical period of time. This allowed us to compare relative expression levels between experimental and control samples. Experimental conditions were carefully maintained between independent assays and analyses were conducted 5–20 independent times. We found it was visually easier to assess expression levels of FOXA2 and SPP1 in the absence of a counterstain; thus, tissue sections were not counterstained. Coverslips were affixed to slides with Permount mounting medium (Fisher Scientific, Ottawa, ON, Canada).

Slides were scanned using an Aperio ScanScope XT in conjunction with the ImageScope software and the area of transverse uterine sections determined using the annotation tool. Total uterine area (including the uterine lumen) and uterine luminal area were calculated and expressed as μ^2^. Uterine luminal area was then subtracted from the total area and the remaining area comprised of the myometrium and endometrium was used as an indication of uterine growth. The data in this study represent the average area (μ^2^) of a transverse uterine section (excluding the uterine lumen) per uterine horn ± SEM. FOXA2 immunostaining was conducted to determine gland number and morphology. The data in this study represent the average number of FOXA2-positive glands/transverse section of uterine horn ± SEM.

### Statistics

The differences between groups were determined using unpaired Student’s *t*-test or one-way ANOVA followed by *post hoc* Student-Newman-Keuls test (GraphPad Prism Software, Inc, La Jolla, CA). All values are expressed as mean ± SEM and a value of P < 0.05 was considered statistically significant.

## Additional Information

**How to cite this article**: León, S. *et al*. Beyond the brain-Peripheral kisspeptin signaling is essential for promoting endometrial gland development and function. *Sci. Rep.* **6**, 29073; doi: 10.1038/srep29073 (2016).
