# Corrigendum: Beyond the brain-Peripheral kisspeptin signaling is essential for promoting endometrial gland development and function

**DOI:** 10.1038/srep30954

**Published:** 2016-08-19

**Authors:** Silvia León, Daniela Fernandois, Alexandra Sull, Judith Sull, Michele Calder, Kanako Hayashi, Moshmi Bhattacharya, Stephen Power, George A. Vilos, Angelos G. Vilos, Manuel Tena-Sempere, Andy V. Babwah

Scientific Reports 6: Article number: 2907310.1038/srep29073; published online: 07 01 2016; updated: 08 19 2016.

The original version of this Article contained a typographical error in the spelling of the author Daniela Fernandois, which was incorrectly given as Daniela Fernadois. This has now been corrected in the PDF and HTML versions of the Article.

